# Detecting Feeding Problems in Young Children with Autism Spectrum Disorder

**DOI:** 10.1007/s10803-021-04869-1

**Published:** 2021-01-16

**Authors:** M. W. G. van Dijk, M. E. Buruma, E. M. A. Blijd-Hoogewys

**Affiliations:** 1 Department of Developmental Psychology at Heymans Institute for Psychological Research, University of Groningen, Grote Kruisstraat 2/1, 9712 TS Groningen, the Netherlands; 2 INTER-PSY, Verlengde Meeuwerderweg 7, 9723 ZM Groningen, the Netherlands

**Keywords:** Feeding problems, Autism, Young children, MCH-FS, Questionnaire, Age effect

## Abstract

Feeding problems are prevalent in children with ASD. We investigated whether the Montreal Children’s Hospital Feeding Scale (MCH-FS, Ramsay et al. in Pediatrics and Child Health 16:147–151, 2011) can be used for young children with ASD. Participants (1–6 years) were selected from a clinical ASD sample (*n* = 80) and a general population sample (*n* = 1389). Internal consistency was good in both samples. In general, parents of children with ASD reported more feeding problems than those from the population sample. The response patterns on the individual items was highly similar. There was a slight increase in symptoms with age in the population sample, but not in the ASD sample. These results suggest that the MCH-FS can be used in populations that include children with ASD.

## Introduction

Children with ASD form a heterogeneous group, with varying degrees of difficulties in social interaction, social communication, and rigidity, and with varying psychiatric comorbidity (APA 2013). A relation between ASD and feeding problems has been identified early in the history of ASD research (e.g. Kanner, 1943 in Ledford and Gast 2006). A systematic review by Sharp et al. (2013a, b) estimated that children with ASD have a fivefold probability of having feeding problems compared to children without ASD. Roughly 44% to 89% of children with ASD have feeding problems (Cermak et al. 2010; Seiverling et al. 2018). For this reason, it has been argued that the presence of severe, atypical or chronic feeding problems should alert professionals in the direction of underlying ASD (Keen 2008), in children as well as in adults (Dell’Osso et al. 2018). Also, the co-authors of this article report from clinical experience that a-specific symptoms, such as problems with sleeping and eating, are often the first to emerge in young children, in many cases before the onset of more specific symptoms of ASD. This illustrates how both scientific studies and clinical experience suggest that feeding problems are frequently comorbid with ASD.

### Definitions and Symptoms

Systematic reviews on feeding problems in ASD have provided robust evidence of significant feeding problems in this population (Ledford and Gast 2006; Sharp et al. 2013a, b). However, there is currently no consensus on terminology with regard to definitions of feeding problems and their symptoms. Terms that are often used are ‘neophobia’ (systematic rejection of novel food), ‘picky eating’ (low appetite, fussy behavior or sensory problems), ‘feeding disorder’ (cases with nutritional, organic or emotional consequences) and ‘feeding difficulties’ (an umbrella term indicating that there is a problem with feeding of some sort) (see Kerzner et al. 2015). Generally, the terms ‘feeding difficulties’ and ‘feeding problems’ are commonly used to specifically refer to problems in (early) childhood which do not necessarily lead to significant nutritional deficiencies, weight loss, or psychosocial problems for the child (yet).

The most prevalent symptom for children with ASD is *food selectivity* (Cermak et al. 2010; Marí-Bauset et al. 2014), which means that they can be very fastidious about which foods they accept and which they refuse. In a study by Seiverling and colleagues (2018), a sample of children with ASD showed more food selectivity by texture and type than a non-ASD sample with language delays (around 24% compared to below 10% in the non-ASD sample). According to caregivers, selectivity can be triggered by various characteristics of the food, such as texture, color, taste, smell and temperature (Williams et al. 2000). It is also known that a lack of food variety—which is the result of food selectivity—is predominant (Nadon et al. 2011). Food selectivity is evident as early as 15 months and although it generally decreases with age, it can persist into adolescence and even adulthood. Research indicates that many children with ASD also have a preference for food with a softer texture (Schreck et al. 2004). In general, these children prefer sweet or fatty foods and refuse fruit and vegetables (Suarez et al. 2014; Vissoker et al. 2019).

Another category of feeding problems in children with ASD is related to their *feeding behavior.* Parents of children with ASD report examples of disturbing behaviors, such as walking away from the table, whining, and yelling (Matson and Fodstad 2009). Other problems that have been mentioned are throwing or dumping foods, difficulty eating at restaurants, and also aggression and tantrums during eating (Provost et al. 2010). Children with ASD often have rituals around food (Vissoker et al. 2015) and show signs of anxiety (Kim et al. 2000). Eating anxiety can be related to compulsive rituals, avoidance of certain types of food (Twachtman-Reilly et al. 2008), and the insistence on specific methods of food preparation, food types and mealtime rules (Matson and Fodstad 2009; Zandt et al. 2007).

An additional kind of feeding problems that is reported is related to an *atypical way of eating*, which involves gagging (Provost et al. 2010), pica, overeating (Vissoker et al. 2015), rapid eating, vomiting, regurgitation, and rumination (Nicholls and Bryant-Waugh 2009; Seiverling et al. 2018). In general, it should be stressed that although feeding problems are generally more prevalent in children with ASD, their degree, expression and development are very idiosyncratic.

### Causes and Consequences

Several factors have been identified to contribute to the etiology of feeding problems in children with ASD. First, the stereotypical and rigid behavior and interests which are characteristic of the disorder have often been associated with the emergence of feeding problems (Vissoker et al. 2015). For instance, Johnson and colleagues (2014) reported a strong association between parental report of feeding problems and the severity of stereotypical behavior. This may also be connected to specific food preferences and idiosyncratic requests and rules these children often have, with regard to the preparation and presentation of the food and choice for particular commercial brands (Ahearn et al. 2001). Children with ASD often have problems with changes in situations and contexts and have a need for consistency. Because meals and eating situations often vary from day to day, this can be challenging for these children (Kuschner et al. 2015).

A second factor is the existence of sensory processing problems in children with ASD (Nadon et al. 2011; Niedźwiecka et al. 2020; Zobel-Lachiusa et al. 2015). The epidemiological-based study of Jussila and colleagues (2020) reported that the prevalence of sensory abnormalities was almost 54% in a sample of 8-year old children with ASD. Clinic-based studies have provided estimations between 69 and 95% (Baranek et al. 2006; Dellapiazza et al. 2018; Tomchek and Dunn 2007). Epidemiological studies screening for ASD probably have a lower percentage due to a larger group with less severe ASD symptoms than participants in clinical studies (Jussila et al. 2020). In any case, sensory problems are common in children with ASD and can be related to the emergence of feeding problems (Vissoker et al. 2015). This is particularly the case in relation to an increased sensitivity to certain texture of the food, which contributes to increased selectivity (Cermak et al. 2010; Johnson et al. 2014; Lane et al. 2010). For instance, Smith and colleagues (2005) reported that in a sample of children with ASD, children with greater sensory hypersensitivity showed more problems with textures of the food and showed more food refusal than children without problems in sensory processing. A longitudinal study of Suarez et al. (2012, 2014) showed that this relation between sensory processing problems and feeding problems is stable across a 2-year period. Sensory processing problems lead to discomfort during eating which may cause anxiety. Suarez et al. (2014) argue that the stereotypical behavior may stem from an attempt to make the meals more predictable and to reduce anxiety. Smith (2016) investigated the patterns of sensory processing with the Sensory Profile and found six out of eight sections to be predictive of eating behaviors in children with ASD. This included tactile, taste/smell, and movement sensitivity, but also under-responsiveness and low/weak energy, suggesting that the total spectrum of sensory processing abnormalities can be associated with feeding abnormalities.

Third, it has been suggested that problems in social communication also contribute to the development of feeding problems in children with ASD. These children are generally less socially motivated to participate in meals (while mealtimes are typically social in nature), are less sensitive to verbal reinforcement and are less competent to imitate positive feeding habits (Johnson et al. 2014). For instance, Postorino et al. (2015) reported that in a sample of children with ASD, children with more limitations in social communications had more feeding problems than children with fewer of such limitations. The problems in social communications may contribute to disruptive mealtime behavior. It may also be speculated that the combination of processing both sensory and social stimuli at the same time lead to feeding problems. Other contributing factors to the etiology of feeding problems in children with ASD are gross motor and fine motor impairments (Kaur et al. 2018), intellectual disability (Matson et al. 2012), and gastro-intestinal dysfunction, which are all more prevalent in children with ASD than in the general population (Williams et al. 2000).

### Detecting Feeding Problems

Consequences of persistent feeding problems in childhood should not be underestimated. In the general population, these problems increase the risk of malnutrition (such as undernutrition), suboptimal or stunted growth, and developmental and cognitive delays (Sharp et al. 2013a, b). In the case of ASD, the study of Hyman et al. (2012) reported that the majority of children are deficient in fiber, choline, calcium, and vitamin D and K. Kral et al. (2014) reported increased waist circumferences and waist to height ratio, putting children with ASD at risk for being overweight. Also, feeding problems do not only have negative consequences for the children themselves, but also for their parents. Caregivers of children with feeding difficulties show higher levels of parental stress (Jones and Bryant-Waugh 2012; Sharp et al. 2013a, b), including parent–child conflict and parent-spouse stress (Kuschner et al. 2017).

Because of the prevalence of feeding problems in children with ASD and their negative consequences for all concerned, pediatricians and health care professionals should be alerted to detect feeding problems in early childhood in order to timely refer for further diagnosis and treatment. Feeding problems are complex in nature and involve many different aspects—such as oral motor skills, feeding history, and feeding behavior—which should be taken into account for diagnosis and treatment (Sanchez et al. 2015). Diagnostic procedures in clinical practice usually consist of reviewing anamnestic information and other parental reports, and conducting a physical examination and a feeding observation (Arvedson 2008). Without adequate diagnosis and treatment, feeding problems tend to persist throughout childhood (Suarez et al. 2014) and adulthood, and may in some cases lead to eating disorders (Westwood and Tchanturia 2017). Early detection can be accomplished by using a screening instrument based on caregiver-reported feeding problems during routine check-ups in the general population. Many existing questionnaires—such as the *Behavioral Pediatrics Feeding Assessment Scale* (BPFAS; Crist and Napier-Phillips 2001), the *Children*’*s Eating Behavior Questionnaire* (CEBQ; Wardle et al. 2001), and the *Mealtime Behavior Questionnaire* (MBQ; Berlin et al. 2009)—are less suitable for quick identification because of their length. For this reason, the *Montreal Children*’*s Hospital Feeding Scale* (MCH-FS) was developed consisting of only 14 items (Ramsay et al. 2011). The questionnaire is based on the observation that clinical and non-clinical groups show similar behaviors around feeding, but that children with feeding difficulties show these behaviors at a higher frequency (Crist and Napier-Phillips 2001). The questionnaire measures seven main constructs: parental concern, family reactions, compensatory strategies, appetite, mealtime behaviors, oral sensory behavior, and oral motor behavior. The MCH-FS has been validated for French, English and Dutch children, and has been demonstrated to have good sensitivity and specificity (Sanchez et al. 2015). The Dutch normative study on children between the ages of 6 months and 3 years showed that the Cronbach’s alpha was 0.84 for the total score, suggesting a robust internal consistency. Evidence was found for a meaningful latent variable structure with two factors—(1) negative mealtime behaviors and (2) negative causes and consequences—but the high correlation between these two factors suggested that a one-factor solution is sufficient for rapid identification of feeding problems. Earlier studies have investigated the use of the MCH-FS in various clinical groups, such as Down Syndrome, children with premature birth and children with cleft palates. The question is whether this general instrument is also useful for children with other types of problems, such as ASD.

It may be the case that feeding problems in children with ASD manifest themselves differently than in the general population. For instance, these children’s preferences and rigidities are argued to be more persistent than in the general population (Bandini et al. 2010; Schreck and Williams 2006). For this reason, the *Brief Autism Mealtime Behaviorist Inventory* (BAMBI) was developed as a short, standardized questionnaire specifically designed to assess feeding problems in children with ASD (Lukens and Linscheid 2008). The instrument aims to assess symptoms unique to the population, such as ritualistic and repetitive behavior during meals (Schreck and Williams 2006) and sensory feeding problems (Cermak et al. 2010). The 18-item version was shown to reflect three underlying factors: limited variety, food refusal and features of autism (Lukens and Lindscheid 2008). The third factor is scored rarely in typically developing children, making the instrument less suitable for use in the general population. In a more extensive study on the BAMBI, the number of items was reduced to 15 with four underlying factors: food selectivity, food refusal, disruptive mealtime behaviors, and mealtime rigidity (DeMand et al. 2015), and according to the authors, this should make the instrument suitable to be used more broadly as a screening measure. However, since the BAMBI was developed specifically for children with ASD and the 4-factor structure is intended to have clinical utility, it may not be the first choice to be used in the general population.

When inspecting the items of both questionnaires, there seems to be a large overlap between the MCH-FS and the BAMBI with regard to assessing sensory motor problems, food refusal, negative mealtime behavior and selectivity. For this reason, we aimed to address the question of whether the MCH-FS, as a short general screener of feeding problems, is also useful for the detection of feeding problems in children between the ages of 1 to 6 years with ASD.

There are indications that in the general population, symptoms of feeding problems ‘build up’ in early childhood as a function of age. For instance, it has been shown that parents of toddlers report a higher incidence of feeding problems than parents of infants (Wright et al. 2007). The problems tend to exacerbate due to the complex interactions between physical, psychological and social factors (e.g. Field et al. 2003; Lindberg et al. 1996; Rommel et al. 2003). The finding that feeding problems seem to increase with age was also corroborated in the normative study for the Dutch version of the MCH-FS in children between the ages of 6 months to 3 years (Van Dijk et al. 2011). It is unknown whether such an age effect also exists in children with ASD, but it seems that this may be much less the case. For instance, the validation study of the BAMBI by Castro et al. (2019) with a large sample of children between ages 7 and 11 years, did not show any age effect. The effect of age was also non-significant in the study of Leiva-García et al. (2019) on the association between feeding problems and oral health status in children with ASD between the ages of 6 and 18 years. In a study of Allen et al. (2015) in children between ages 2 and 5 years, feeding problems were shown to be associated with ASD symptoms, behavior problems, sleep problems, and parenting stress, but not with child age. In contrast, some studies did report age effects. For instance, in the study of Nadon et al. (2011) in which children aged 3 to 12 years with ASD were compared with their typically developing peers, older children had fewer problems than younger children. In addition, the study by Beighley et al. (2013) reported a slight downward trend for food selectivity severity across childhood for a combined sample of children with different levels of ASD severity. The study by Vissoker et al. (2015) showed that children with ASD between 3 and 7 years of age showed more ritualistic behavior around food than children between 2 and 3 years. However, other eating problems (such as chewing and swallowing problems, avoidance, and food selectivity) did not show any age-related differences. In conclusion, although only few studies address age-related changes in feeding problems in children with ASD, there is some evidence suggesting that the effect of age in children with ASD is marginal or small compared to that in typically developing children. The existence of such systematic age differences is relevant for the use of any general screening instrument. If certain problems occur more frequently in any specific age group, it may indicate that these are to some extent developmentally appropriate and transient. Previous studies suggest that feeding problems in typically developing children are somewhat more prevalent around the age of 2 and 3 years. The question is whether such a trend is also visible in the development of children with ASD. Considering the various differences between the two groups relevant to eating characteristics, this is not expected to be the case.

Because of the prevalence of feeding problems, healthcare providers are encouraged to include screening of feeding problems as part of routine medical examinations, both in the general population of young children (Ramsay 2011) and children with ASD (Sharp et al. 2013a, b). The aim of the current study is to evaluate the use of the MCH-FS in a sample with ASD.

### Research Questions

1. What are the psychometric properties of the MCH-FS in a sample of children diagnosed with ASD compared to children from the general population?
2. Do children with ASD show more symptoms of feeding problems than children from the general population as measured with the MCH-FS?
3. Do children with ASD show a difference on individual symptoms of feeding problems as measured on the MCH-FS?
4. Is there a relation between reported feeding problems and age throughout early childhood for children with ASD and children from the general population?

## Method

### Participants

Participants were the caregivers of two groups of children. The first group (*n* = 80, 55 boys, 25 girls) consists of a *clinical sample* of caregivers of children between the ages of 1 and 6 years (*M* = 207 weeks, *SD* = 57.69) who were diagnosed with ASD by the young children expert team at INTER-PSY, a large mental health care institution in the North of the Netherlands. This expert team is specialized in diagnosis and treatment of young children (0–6 years) with a wide variety of developmental and psychiatric problems (such as ASD, ADHD, ODD, trauma and parent–child interaction problems). They follow the Dutch clinical best estimate for ASD diagnostics in young children (Blijd-Hoogewys et al. 2017). In the clinical sample, there was one participant aged 13.5 months, who received the ASD diagnosis at a later point in time. All other participants were 18 months of age or older. It has been generally accepted that it is possible to reliably diagnose ASD from the age of 18–24 months onwards (Zwaigenbaum et al. 2016). Indeed, the ASD diagnosis of all children in this study was later confirmed after psychiatric reevaluation. Children in the clinical sample had a mean total score of 14.75 (SD = 5.00) on the ADOS-2 (the cut-off score for diagnosing ASD is 7). The cognitive development of these participants at time of inclusion was slightly below average (IQ/developmental index on either the WPPSI, SON-R or Bayley Developmental Scales (see below) was M = 90.32 (SD = 18.25). The data was collected from 2014 to 2017.

The second group of participants consists of caregivers from the *general population sample* (*n* = 1389). Children between the ages of 1 and 3 years were taken from the original dataset of the MCH-FS normative study, as published in Van Dijk and colleagues (2011). A normative sample of 965 caregivers of children between the ages of 1 and 3 years (*M* = 119.48 weeks, *SD* = 48.49 weeks) living in the province of Groningen, the Netherlands (including (sub) urban and rural areas), participated in the study when visiting their local Infant Welfare Center (‘Consultatiebureau’ in Dutch). For the purpose of the current study, an additional sample (*n* = 427) was collected (in 2018) of caregivers of older children in order to be able to compare with the full age range of the clinical sample. They were between the age of 4 to 6 years (*M* = 281.83 weeks, *SD* = 46.67 weeks). The total general population sample of 1389 children consisted of 706 boys and 676 girls (in 7 cases, ‘sex’ was left blank). The mean age was 169.38 weeks (*SD* = 88.96 weeks).

### Measures

The Dutch version of the MCH-FS (called the *Screeninglijst Eetgedrag Peuters*/SEP; see Van Dijk et al. 2011) was used to assess severity of feeding problems. The caregiver was asked to rate each of the items on a 7-point scale. The questionnaire has anchors on both extreme positions, but no labels for values between 2 and 6 (for instance, item 2 ‘How worried are you about your child’s eating?’ goes from ‘not worried’ at value 1 to ‘very worried’ at value 7). The scores on items numbers 1, 3, 4, 8, 10, 12 and 13 had to be inverted, so that high values always indicate a greater severity of symptoms.

In some cases, the questionnaire was not filled in by participants as instructed. When informants marked two neighboring values (for instance, ‘1’ and ‘2’), we used the highest value (‘2’, in the example). Some caregivers did not fill in item 4 (‘When does your child start to refuse food?’) but wrote behind the item ‘does not refuse’. This was interpreted as the extreme anchor point ‘at the end of a meal’. The total score was computed by adding up all values. Percentile cut-off points of 84.1, 95.0 and 97.5 represent ‘mild’, ‘moderate’ and ‘severe’ problems, respectively.

### Procedure

Caregivers in the *general population sample* with a child between the ages of 1 and 3 year filled out the Dutch version of the MCH-FS when they were visiting their routine check-up at the local Child Health Center in 2011. The data were collected with paper-and-pencil and were imported to SPSS manually. For the purpose of the current study, caregivers of children between the ages of 4 and 6 years were recruited by a research panel in 2018. These participants filled out the MCH-FS using an online version of the questionnaire in Qualtrics with a page layout as similar as possible to the original layout.

The data from the *clinical ASD sample* was based on retrospective chart review. Here, caregivers had filled out the MCH-FS as part of the regular diagnostic trajectory of their child (between February 2014 and August 2017). This was usually done in the 2–3 weeks before the intake procedure. The diagnostic trajectory took roughly 6–8 weeks in total and consisted of reviewing anamnestic information, observations by a pediatrician, home observation and observation in a second context, a cognitive test (Bayley-III-NL, van Baar et al. 2014; WPPSI-III-NL, Hendriksen and Hurks 2009; SON-R 2.5-7, Tellegen et al. 1998; or SON-R 2-8, Tellegen and Laros 2017), and the ADOS-2 (de Bildt et al. 2013). For the current data set, only children who were diagnosed with ASD were included in the study.

Ethical approval of the Ethical Committee of Psychology was granted for all three parts of the study. Data analysis consisted of scale analysis and—because of highly skewed and distributions with different characteristics for both groups—consecutive non-parametric testing. First, internal consistency was assessed using Cronbach’s α and Guttman’s λ-2 for both groups separately. In addition, the item score ranges, means, standard deviations and skewness’ were computed for both the total MCH-FS score and the scores on the individual items. The differences between the scores of the normative sample and the clinical ASD sample, both for the total score and the individual items, were tested by means of Mann–Whitney U tests. A Bonferroni correction for multiple comparisons was made with statistical significance accepted at the *p* < 0.0033 level. After this, we tested whether children of different ages scored differently on the MCH-FS, for both groups separately with a Kruskall-Wallis H test and post hoc pairwise Mann–Whitney U tests. For testing the group difference, we used an alpha of 0.05. A Bonferroni correction was applied for the multiple post hoc contrasts testing. The analyses were performed in SPPS, and the figures were made in Excel and R.

## Results

### Psychometric Properties

The results demonstrate a good internal consistency for the items of the Dutch version of the MCH-FS for both the general population sample (α = 0.847, Guttman’s λ-2 = 0.856) and for the clinical ASD sample (α = 0.828, Guttman’s λ-2 = 0.839). For both groups, the Cronbach’s alpha increased slightly (to 0.848 and 0.839 respectively) when removing item 5 (‘How long do mealtimes take for your child?’).

Table 1 describes the mean, standard deviation, empirical range and skewness for each item for both groups separately. The results show that for most items, the values of the clinical ASD sample seem to be higher than the general population sample, which indicates more feeding problems. The standard deviations of both groups are in the same order of magnitude (above or close to 1 for all items). Participants use the full theoretical score range between 1 and 7 for all items in the general population sample, and for 11 items in the clinical ASD sample (scores on items 10, 11 and 13 are between 1 and 6). The results show that all items are non-normally distributed for both groups, and in most cases positively skewed.

**Table 1:** Descriptive statistics for the MCH-FS items for the general population sample and the clinical ASD sample

MCH-FS	General population sample				Clinical ASD sample			
Item content	M	SD	Range	Skewness	M	SD	Range	Skewness
1. Difficult mealtimes	2.84	1.38	1–7	1.38	3.99	1.47	1–7	0.00
2. Worries about feeding	2.19	1.45	1–7	1.45	2.67	1.72	1–7	0.83
3. Poor appetite	3.25	1.17	1–7	1.17	3.47	1.29	1–7	0.13
4. Start refusing food	3.48	2.00	1–7	2.00	4.90	2.06	1–7	− 0.62
5. Long mealtimes	2.77	0.97	1–7	0.97	2.44	1.28	1–7	1.12
6. Bad behavior	2.86	1.48	1–7	1.48	3.85	1.56	1–7	− 0.22
7. Gags/spits/vomits	1.72	1.20	1–7	1.20	2.26	1.81	1–7	1.26
8. Holding food in mouth	2.09	1.49	1–7	1.49	2.45	1.80	1–7	1.05
9. Follow around/distract	2.33	1.65	1–7	1.65	3.01	2.10	1–7	0.62
10. Force to eat	2.46	1.60	1–7	1.60	2.84	1.65	1–6	0.46
11. Poor chewing	1.63	0.97	1–7	0.97	1.87	1.16	1–6	1.49
12. Poor growth	1.71	1.16	1–7	1.16	1.66	1.17	1–7	2.21
13. Influence relation	1.75	1.13	1–7	1.13	1.96	1.20	1–6	1.17
14. Influence family relations	1.99	1.44	1–7	1.44	2.50	1.78	1–7	0.91
Total score	33.07	11.26	15–73	11.26	39.96	12.52	18–67	0.31

### Differences Between the General Population Sample and the Clinical ASD Sample

**Fig. 1:**
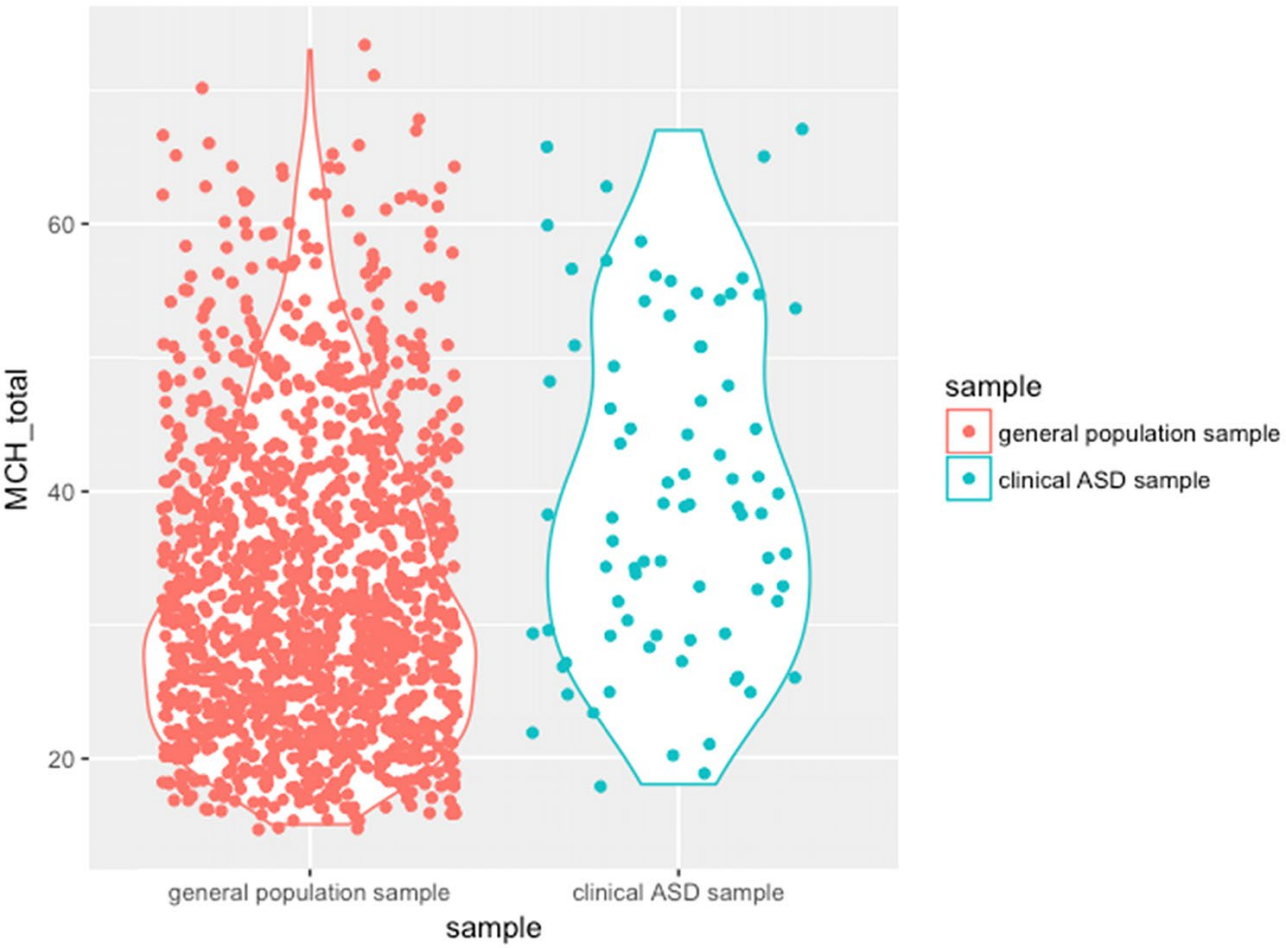
Violin plots of the distribution of scores in both the clinical ASD sample and the general population sample

A Mann–Whitney U test indicated a significant difference between the total score on the MCH-FS for the general population sample and the clinical ASD sample. Distributions of the total scores were not similar, as assessed by visual inspection. The mean rank for general population sample (722.19) and the clinical ASD sample (957.48) was statistically significantly different (*U* = 73.358, *z* = 4.826, *p* < 0.001). Generally, caregivers of a child with ASD report more symptoms of feeding problems than caregivers of a child from the general population. In addition, the violin plot presented in Fig. 1 shows that the distribution of scores in the clinical ASD sample seemed to be much less positively skewed than in the general population sample.

When considering the total score on the MCH-FS of the children with ASD in relation to the 84.1, 95.0 and 97.5 percentile cut-off point calculated in the normative sample (representing ‘mild’, ‘moderate’ and ‘severe’ problems, respectively), 15 children fell in the category ‘mild problems’, 6 children in the category ‘moderate problems’, and 3 children in the category ‘severe problems’.

**Fig. 2:**
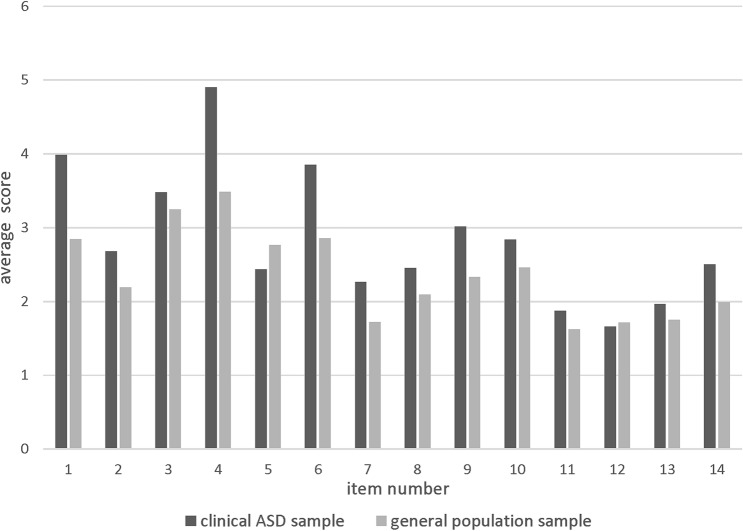
Bar graph of average scores on the items of the MCH-FS for both the clinical ASD sample (ASD) and the general population sample

When inspecting the scores on the individual items of the MCH-FS (see Fig. 2), Mann–Whitney U tests revealed significant differences between groups for items 1 (*U* = 78.895, *z* = 6.484; *p* < 0.001), 4 (*U* = 76.597, *z* = 5.773; *p* < 0.001), 5 (*U* = 43.592 *z* = − 3.469; *p* = 0.001), and 6 (*U* = 75.377, *z* = 5.483; *p* < 0.001). With the exception of item 5 (‘How long do mealtimes take for your child?’), the clinical ASD sample has a higher score on each of the items. The difference between the two groups is largest for item 1 (‘How do you find mealtimes with your child?’), 4 (‘When does your child start refusing to eat during mealtimes?’) and 6 (‘How does your child behave during mealtimes?’). When inspecting the average values for each item in both samples, there seemed to be a similar response pattern: items that are scored relatively high in the general population sample are also scored relatively high in the clinical ASD sample.

### Age Differences

A Kruskal–Wallis H test was conducted to determine if there were differences in MCH-FS scores between age groups (1, 2, 3, 4, 5 and 6 years). For the general population sample, median MCH-FS scores were statistically significantly different between the different age group (χ^2^(5) = 138.282; *p* < 0.001). Additional post-hoc Mann–Whitney U tests demonstrated that within the general population sample, the 2-, 3-,4-, 5-, and 6-year olds scored higher than the 1-year olds (all *p* < 0.001), the 4-year olds scored higher than the 2-year olds (*p* = 0.001).

**Fig. 3:**
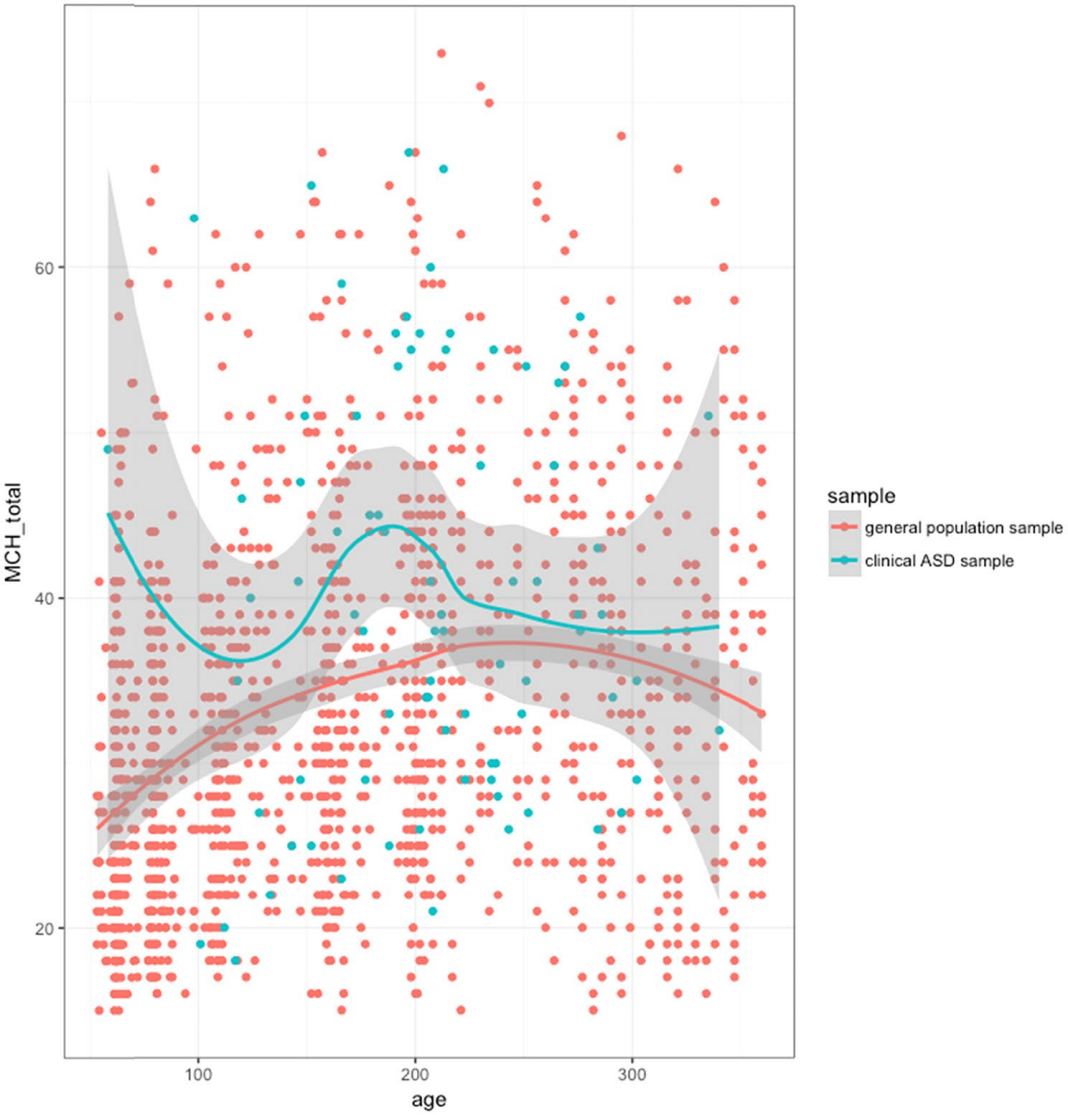
Total scores on the Dutch version of the MCH-FS in relation to the participant’s age (data points and Loess trend with 95% confidence interval)

Figure 3 however, shows a (slight) reversed U-shaped trend for the general population sample. This suggests a slow built-up of problems till the age of 4, after which a mild decrease sets in. However, such a pattern was not observed in the children with ASD. For the clinical ASD sample, median MCH-FS scores were not statistically significantly different between the different age group (χ^2^(5) = 4.015, *p* < 0.547).

## Discussion

Because of the prevalence of feeding problems in early childhood and their associated negative consequences for child development and parent’s wellbeing, it has been argued that clinicians should be alert to detect these problems early on in life. A screening instrument based on caregiver-reported feeding problems—such as the Montreal Children’s Hospital Feeding Scale (MCH-FS)—can be used for the quick and early identification of such problems in the general population.

The results of the current study show that the Dutch version of the MCH-FS—called the SEP—has a good internal consistency in both the general population sample and the (clinical) ASD sample. The response patterns showed adequate use of the theoretical score range, and (slight) positively skewed distributed answer patterns as is to be expected for a screening instrument (Counsell et al. 2011). In addition, caregivers of children with ASD reported more symptoms of feeding problems on the instrument compared to caregivers in the general population. They also showed a similar global response pattern on the individual items of the MCH-FS, in the sense that for most items they scored higher than the general population sample. The only exception was item 5 (‘How long do mealtimes take for your child?’) which indicated that children with ASD have shorter meals than children from the general population. This underscores the observation that rapid eating has been reported to be a common feeding problem in individuals with developmental disorders (Anglesea 2008) and is specifically reported often in children with ASD (Seiverling 2010). Rapid eating has also been associated with co-morbid psychopathology and emotional difficulties in children with ASD (Leader et al. 2020). Short meals can also stem from the fact that children with ASD often have limited attention skills (Dellapiazza et al. 2018; Lyall et al. 2017), which may lead them to lose interest in eating earlier than their non-ASD peers. It is important to note that previous analyses on the MCH-FS in the general population had already shown that the item on mealtime duration was psychometrically problematic in the sense that it did not belong to any of the two underlying factors; it had a very low item-total correlation (Van Dijk et al. 2011). Notably, it measures a non-linear underlying scale because both very long and very short meals can be symptomatic of feeding problems. The current study showed that item 5 is even more problematic for children with ASD, as they generally score lower on this item, which may cause an underestimation of the total amount of feeding problems. This suggests that—although information about mealtime duration in children with ASD may be valuable in a qualitative sense—the score on this item should not be included in the feeding problems total score. Taking out item 5 also improves internal consistency, but only slightly. For all MCH-FS items aside from item 5, caregivers of a child with ASD reported higher scores, indicating greater problems. This was particularly the case for items with the underlying variables ‘difficult mealtimes’, ‘food refusal’ and ‘problematic mealtime behavior’. This suggests that children with ASD show behaviors that are also observed in the general population, but at a higher rate. This higher rate was also expected on the basis of the literature reporting that feeding problems in children with ASD are more common than in the general population (as reported by Seiverling et al. 2018).

When inspecting the distributional characteristics of the samples, two other differences between groups could be observed. First, although the general population sample showed a slight increase in reported symptoms till the age of 4, after which a slight decrease sets in, this trend was not observed in the clinical ASD sample. This is consistent with previous literature by Allen et al. (2015) that reported that feeding problems in children with ASD between ages 2 and 5 years, were related to all kinds of variables but not to child age. In addition, Vissoker et al. (2015, 2019) showed that children with ASD aged 3–7 years exhibited more ritualistic behavior around food than children aged 2–3 years (marginal significance) but that other eating problems such as commonly observed behaviors like food avoidance and food selectivity did not show any age-related differences. Ritualistic behavior is part of rigidity, which often emerges later in the development of children with ASD (Steiner et al. 2012). This suggests that the development of feeding problems in this population emerge as part of other ASD characteristics which become more notable as developmental tasks become more complex over time. However, although some authors have reported on age-related differences earlier, these results were not consistent across studies.

In addition, the current study did not find any evidence supporting the hypothesis that age-related changes in feeding problems exist in children with ASD. Second, the distribution of scores seemed to be less positively skewed in the clinical ASD sample than in the general population sample. This may be a result of the larger prevalence of feeding problems in children with ASD, as stated previously. Whereas in the majority of the general population children have no or only minor symptoms, this is much less the case for children with ASD. It is important to note that both groups do use the total score range to a similar degree, suggesting that the MCH-FS is a similar measure to differentiate between no, mild and major feeding problems in both groups.

We conclude that the MCH-FS can be used in a sample of children that includes children with ASD. The results of this study showed no indications that the use of this instrument for a sample that includes children with ASD would be problematic. Both item pattern and psychometric properties are highly similar in children between the ages of 1 and 6 years with ASD and children from the general population. Children with ASD score higher, evidencing more feeding problems, as was expected. Our results are consistent with those of Dovey et al. (2020) which revealed more similarities than differences in eating problems between typically developing children and children with ASD, ARFID (Avoidant/Restrictive Food Intake Disorder) and picky eating. The fact that the sample of children in our study with ASD did not show the age-related differences may indicate that feeding problems are less transient in children with ASD, which may possibly relate to these children’s behavioral rigidity. However, this would not explain why feeding problems first increase in the general population sample until the age of 4 years, only after which a decrease sets in.

Further research is needed on the sensitivity and specificity of the instrument in this group, for instance by comparing the scores on the MCH-FS with scores on the BAMBI and the assessment by a practitioner. The main advantage of the MCH-FS is the fact that it is a very short instrument (only 13 items after removing item 5) that still covers the main symptoms of feeding difficulties, and that it is not specific for (any type of) typically or atypically developing children. For this reason, it is very well suited to function as a general screening instrument as a first step in the diagnostic process. If significant feeding problems are found in children with ASD, it may be advised to use a more specific instrument focusing on more specific symptoms in this subgroup, such as the BAMBI and by collecting additional information from parents.

Because feeding problems in early childhood have clear aversive consequences for child development and family wellbeing, early detection is important. For instance, Ledford and Gast (2006) argue that even when the health of the child is not immediately at risk, assessment of problematic feeding behaviors in the population of children with ASD should be a priority. More generally, Sharp, Berry and colleagues (2013) claim that there is a clear need for a frontline feeding screening tool that can be used during medical appointments. The results so far suggest that the MCH-FS might serve such a function and might make early detection of feeding problems easier. This can lead to more timely help and intervention for caregivers. This is necessary because family meals including children with feeding problems are described as exceptionally stressful (e.g. Garro et al. 2005)*.* In addition, caregiver-child interactions are often mentioned as a contributing or maintaining factor in the persistence of the feeding difficulty (Berlin et al. 2009; Piazza et al. 2003), often because of (unintentional) forms of positive and negative reinforcement that take place (Bachmeyer et al. 2009). This may be especially important for caregivers of children with ASD because they significantly more often prompt and encourage children to eat, compared to typically developing children (Kral et al. 2014) which may lead the feeding problems to exacerbate over time.

There are some clear limitations of this study. First of all, there are obvious limitations with regard to the sample of children with ASD. Although the size of this sample was substantial (*n* = 80)—especially considering the fact that they are of a young age—it was small relative to the sample from the general population (*n* = 1389). It is possible that this smaller sample size is (partly) responsible for the fact that the effect for child age could not be established. However, this is not reflected in the distribution of scores in relation to age, where not even the slightest trend with regard to age can be detected. This means that the lack of a trend may not be just a matter of lack of statistical power. There was another difference between the two samples, namely the boy-to-girl ratio. Since ASD is generally more prevalent in boys, the clinical sample was not distributed equally between the sexes. However, in our clinical sample, there was a boy-to-girl ratio of 2:1. This means that we were able to include a relatively large number of girls for this study, which is quite unique. The traditional boy-to-girl ratio is 4:1; however, this ratio is considered to be an underestimation (Loomes et al. 2017). A second limitation in the study is that the use of parental report relies on information that may be prone to bias. Also, we did not focus specifically on food selectivity but on the umbrella term of ‘feeding problems’. This means that the screening instrument does not focus on one of the most distinctive symptoms of feeding problems for children with ASD. Though the MCH-FS has an advantage concerning its usefulness for a large variety of typically and atypically developing children, it may not be the best instrument to investigate age-related changes of feeding problems in a sample of young children with ASD. Future research should be directed at targeting this specific question. For the usefulness of the MCH-FS, the lack of the age effect for children with ASD does not carry immediate consequences since children with ASD have a higher score on average for all age-related norm groups. Construction of different norm groups for this specific population would lead to a decrease of sensitivity which would be undesirable for a screening instrument.

This cross-sectional study was a first attempt to compare age-related changes in feeding problems in both a clinical sample of children with ASD and a general population sample using the MCH-FS as a screening instrument. The suggestion that feeding problems in children with ASD may be less transient then in children from the general population is important and should be addressed in future longitudinal research. Particularly the speculation that there is a complex interaction between the development of feeding problems, ritualistic behaviors, sensory issues and other characteristics of ASD deserves further attention.
